# Renal ROCK Activation and Its Pharmacological Inhibition in Patients With Diabetes 

**DOI:** 10.3389/fphar.2021.738121

**Published:** 2021-09-07

**Authors:** Keiichiro Matoba, Kensuke Sekiguchi, Yosuke Nagai, Yusuke Takeda, Hiroshi Takahashi, Tamotsu Yokota, Kazunori Utsunomiya, Rimei Nishimura

**Affiliations:** ^1^ Department of Internal Medicine, Division of Diabetes, Metabolism, and Endocrinology, The Jikei University School of Medicine, Tokyo, Japan; ^2^ Center for Preventive Medicine, The Jikei University School of Medicine, Tokyo, Japan

**Keywords:** Rho-kinase (ROCK), diabetic nephropathy, chronic kidney disease, diabetes, cell signaling, fasudil

## Abstract

Rho-associated coiled-coil-containing protein kinase (ROCK) is a serine/threonine kinase with essential roles in cytoskeletal functions. Substantial evidence implicates ROCK as a critical regulator in the inception and progression of diabetic nephropathy through a mechanism involving mesangial fibrosis, podocyte apoptosis, and endothelial inflammation. Despite these experimental observations, human data is lacking. Here we show that the phosphorylated form of myosin phosphatase targeting subunit 1 (MYPT1), a ROCK substrate, was increased in both the glomerular and tubulointerstitial areas in patients with histologically confirmed diabetic nephropathy. We also conducted a retrospective pilot analysis of data from patients with diabetes to assess the renoprotective effects of fasudil, an ATP-competitive ROCK inhibitor licensed in Japan for the prevention of vasospasm following subarachnoid hemorrhage. Fifteen subjects (male, *n* = 8; female, *n* = 7; age 65.7 ± 14.7 years; body height, 161.1 ± 12.6 cm; body weight, 57.6 ± 13.7 kg; body mass index, 22.4 ± 3.7 kg/m^2^) were enrolled to evaluate blood pressure and the renal outcome after fasudil treatment. Of note, proteinuria was significantly reduced at the end of the fasudil treatment without affecting the blood pressure or estimated glomerular filtration rate. Taken together, these findings suggest that the administration of fasudil could be associated with a better renal outcome by inhibiting the ROCK activity in patients with diabetes.

## Introduction

Diabetic nephropathy is a major health concern that imposes a significant risk for end-stage renal disease. A series of clinical trials demonstrated significant improvement in renal outcomes of patients with diabetic nephropathy after long-term treatment with sodium glucose co-transporter 2 inhibitors (Wanner et al., 2016), mineralocorticoid receptor antagonists (Lytvyn et al., 2019), endothelin receptor antagonists (Cahn et al., 2019), or Janus kinase-signal transducer and activator of transcription (JAK-STAT) inhibitors (Brosius et al., 2016). In addition, experimental studies revealed the protective actions of SIRT3 (Srivastava et al., 2018; Srivastava et al., 2021a), fibroblast growth factor receptor 1 (Li et al., 2020), and glucocorticoid receptor in endothelium (Li et al., 2020; Srivastava et al., 2021b), as well as N-acetyl-seryl-aspartyl-lysyl-proline, an antifibrotic peptide (Kanasaki et al., 2014; Srivastava et al., 2020; Srivastava et al., 2021b). However, the progression of diabetic nephropathy is still associated with high mortality and morbidity rates, even when patients receive the current standard of care (de Zeeuw and Heerspink, 2016; Waijer et al., 2020; Heerspink et al., 2021). Thus, the elucidation of novel, clinically translatable targets is required.

Rho-associated coiled-coil-containing protein kinase (ROCK) is a serine/threonine kinase that was originally identified as RhoA interacting protein. A wide range of cellular programs (e.g., cytoskeletal remodeling, cellular migration) are unleashed by ROCK activation via phosphorylation of myosin phosphatase target subunit 1 (MYPT1) (Fujisawa et al., 1996; Uehata et al., 1997). Experimental studies have demonstrated that the phosphorylated form of MYPT1 is increased in the renal cortex of both type 1 and type 2 diabetes rodent models (Peng et al., 2008; Matoba et al., 2013), indicating that ROCK activity is enhanced in the diabetic kidney, regardless of the type of diabetes. We and others have reported that ROCK induces mesangial expansion (Matoba et al., 2014), podocyte apoptosis (Matoba et al., 2013), and endothelial injury (Shimokawa, 2002; Takeda et al., 2019) by producing inflammatory cytokines, adhesion molecules, and oxidative stress. Besides, ROCK plays a key role in the process of vascular smooth muscle contraction and therefore the pathogenesis of vascular spasm (Masumoto et al., 2002; Wickman et al., 2003).

Fasudil hydrochloride is the clinically-approved ROCK inhibitor for the short-term treatment of cerebral vasospasm as an IV regimen. It has been licensed in Japan since 1995, and several thousand patients with subarachnoid hemorrhage have been treated with fasudil for this indication without major safety concerns (Suzuki et al., 2008; Zhao et al., 2011). In addition, clinical trials for other applications—most frequently for cardiovascular disease including cardiac vasospasm and pulmonary hypertension—have been performed (Vicari et al., 2005). Initial insights linking ROCK to diabetic nephropathy were gleaned from studies that assessed the effect of fasudil on streptozotocin-injected type 1 diabetic rats (Gojo et al., 2007). In this model, the pharmacological inhibition of ROCK was effective for the prevention of albuminuria and glomerular sclerosis via the attenuation of oxidative stress. More recently, we provided the inaugural evidence implicating ROCK as a core component of the transcriptional circuitry that governs inflammation and hypoxic reactions in type 2 diabetic db/db mice (Matoba et al., 2013; Nagai et al., 2019). These findings indicate that ROCK may be involved in the major pathogenesis of diabetic renal damage and a promising therapeutic target to prevent the progression of diabetic nephropathy. However, the renal ROCK activity in humans has never been explored and the renoprotective effects of ROCK inhibition has not been investigated in patients with diabetes.

In the present study, we assessed the activity of ROCK in the kidneys of patients with diabetic nephropathy and conducted a retrospective observational study in patients with diabetes to evaluate the acute clinical effects of intravenously administered ROCK inhibitor on the renal outcome.

## Methods

Histology. Formalin-fixed paraffin-embedded human specimens were obtained from the ProteoGenex and stored at 4°C until use. Human sample collection was performed under approval of the Ethical Committee. In total, 8 postmortem kidney slices were examined, including 4 control kidney slices from healthy donors and 4 from patients with diabetic nephropathy. The patients’ age at the time of death, sex, body mass index, postmortem interval, pathological diagnosis, cause of death, and comorbidities are described in Supplemental Table S1. Diabetic nephropathy was histologically confirmed by hematoxylin and eosin staining. Criteria for diabetic nephropathy included glomerular hypertrophy, diffuse mesangial and focal nodular glomerulosclerosis.

For immunofluorescence of glomeruli and tubulointerstitium, 4-μm-thick paraffin-embedded sections were deparaffinized and subjected to antigen retrieval by heating in citrate buffer for 40 min. Endogenous peroxidase activity was blocked by the treatment with 3% hydrogen peroxide for 5 min. Sections were incubated overnight at 4°C with anti-phospho-MYPT1 Thr853 (p-MYPT1) antibody (1:50 dilution, Thermo Fisher Scientific, Catalog No. PA5-38299, Lot No. SG2414321A). After incubation with biotinylated antibody (BioGenex Laboratories, Catalog No. HK595-50K, Lot No. HK5950618A) for 15 min, the sections were finally developed with Super Sensitive DAB (BioGenex Laboratories, Catalog No. B-HK542XAK). Image acquisition was performed using an EVOS M5000 Imaging System (Thermo Fisher Scientific) and analyzed using the ImageJ software program (NIH). The analyzer was blinded to the identity of each section. In glomeruli, the positive area was determined as the ratio of the p-MYPT1 stained area to the glomerular tuft area in 20 randomly selected glomeruli cut at their vascular pole. The tubulointerstital p-MYPT1-positive area was quantified in randomly selected 10 non-overlapping fields to determine the percentage of the positively stained area to the total tissue area. A negative control image stained with rabbit immunoglobulin fraction (Dako, Catalog No. X0903, Lot No. 20059712) is shown in Supplemental Figure S1A. Representative images of H&E-stained glomeruli are presented in Supplemental Figure S1B. The expansion of glomerular surface area, traced in 10 randomly selected non-overlapping fields, was not statistically significant in these patients.

Patients. The use of human data was approved by the Ethics committee of the Jikei University School of Medicine (No. 30–271, 9292) and the research conformed to the Declaration of Helsinki. The medical records of patients with diabetes who visited Jikei University Hospital, Jikei University Katsushika Medical Center, Jikei University Daisan Hospital, and Jikei University Kashiwa Hospital, between January 2016 and October 2018 were retrospectively reviewed. Diabetes was defined as the use of diabetes medication during this period. Among 23,241 subjects with diabetes, 15 patients were treated with fasudil for the prevention of vasospasm after cerebral hemorrhage. From a total of 15 patients, 12 patients were treated with insulin, the remaining 3 received oral treatment. We excluded patients whose records were missing data from urinalysis pre- or post-treatment of fasudil from the analysis of proteinuria. A urine dipstick analysis was used to detect proteinuria. Proteinuria 15 mg/dl and 30 mg/dl was graded as +1 and +2, respectively.

Statistical analyses. Data are represented as the means, and error bars depict the s.e.m. (*n* as indicated in the figure legends). The measurements were taken from distinct samples. Statistical evaluations of two groups were performed using a two-tailed Student’s *t*-test, with the exception of the urinary dipstick test data, which were compared using the Mann-Whitney U test. *p* values of <0.05 were considered to indicate statistical significance.

## Results

### Renal ROCK Activity is Elevated in Patients With Diabetic Nephropathy

**FIGURE 1:**
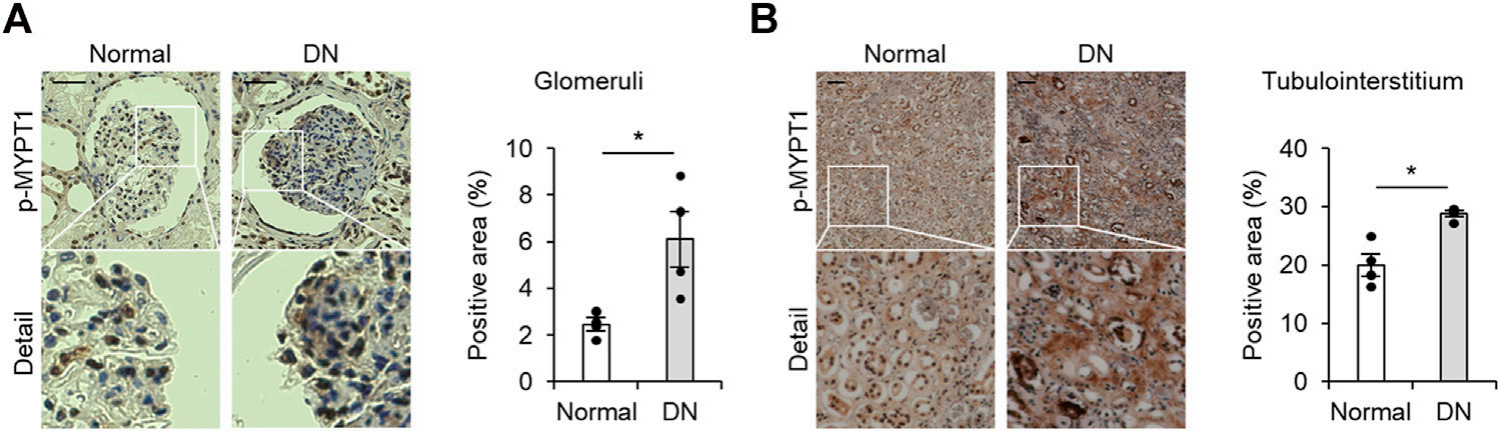
Activated ROCK signaling in patients with diabetic nephropathy **(A)** Representative images of p-MYPT1 staining in the glomeruli of healthy donors and patients with diabetic nephropathy (DN). The glomerular-positive area was calculated as the ratio of the p-MYPT1-positive area to the glomerular tuft area. The scale bar on the top left presents 50 μm p-MYPT1, phosphorylated form of MYPT1. **(B)** Representative images of tubulointerstitum stained with an antibody against p-MYPT1 (n = 4). The percentage of the p-MYPT1-positive area in the kidneys was evaluated. The scale bar on the top left presents 100 μm *p < 0.05. Data represent the mean ± s.e.m.https://www.frontiersin.org/about/author-guidelines.

To gain insights into the potential role of ROCK in the human diabetic kidney, we first assessed the renal activity using an antibody that detects MYPT1 phosphorylated at Thr853, a ROCK target. As shown in Figure 1A, high levels of phosphorylated MYPT1 were visualized in the glomerular area obtained from patients with histologically proven diabetic nephropathy in comparison to kidney tissue of healthy donors. In addition, the tubulointerstitial area that was positive for p-MYPT1 was significantly increased in patients with diabetic nephropathy (Figure 1B). These immunohistochemical analyses provided evidence suggesting increased renal ROCK activity in human diabetic nephropathy.

### Fasudil Does Not Affect Blood Pressure in Patients With Diabetes

As the above results suggested that ROCK activity was increased in the human diabetic kidney, we next sought to test whether a ROCK inhibitor could ameliorate renal dysfunction in patients with diabetes. We performed a retrospective pilot study from a cohort of 15 patients with diabetes (male, *n* = 8; female, *n* = 7; age 65.7 ± 14.7 years; body height, 161.1 ± 12.6 cm; body weight, 57.6 ± 13.7 kg; body mass index, 22.4 ± 3.7 kg/m^2^) who administered fasudil for the prevention of vasospasm following subarachnoid hemorrhage, focusing on the association between ROCK inhibition and the renal outcomes. The baseline characteristics of patients are listed in Table 1. In this analysis, diabetes was defined by the use of glucose-lowering medications.

**TABLE 1:** The baseline characteristics of patients with diabetes treated with fasudil. LDL-C, low destiny lipoprotein cholesterol; HDL-C, high lipoprotein cholesterol TG, triglyceride; ARB, angiotensin II receptor blocker; αGI, α-glucosidase inhibitor, DPP-4, dipeptidyl peptidase IV.

Number (male/female)	15 (8/7)
Age (years)	65.7 ± 14.7
Height (cm)	161.1 ± 12.6
Body weight (kg)	57.6 ± 1.07
Body mass index (kg/m^2^)	22.4 ± 3.7
Blood glucose (mg/dL)	174.7 ± 44.0
HbA1c (%)	6.6 ± 1.4
Uric acid (mg/dL)	3.6 ± 1.9
LDL-C (mg/dL)	128.0 ± 35.8
HDL-C (mg/dL)	54.6 ± 17.3
TG (mg/dL)	114.2 ± 71.9
ARBs (%)	45.5
Sulfonylurea (%)	0.0
αGI (%)	6.7
Metformin (%)	6.7
Thiazolidness (%)	0.0
DPP-4 inhibitor (%)	6.7
Statin (%)	6.7

**FIGURE 2:**
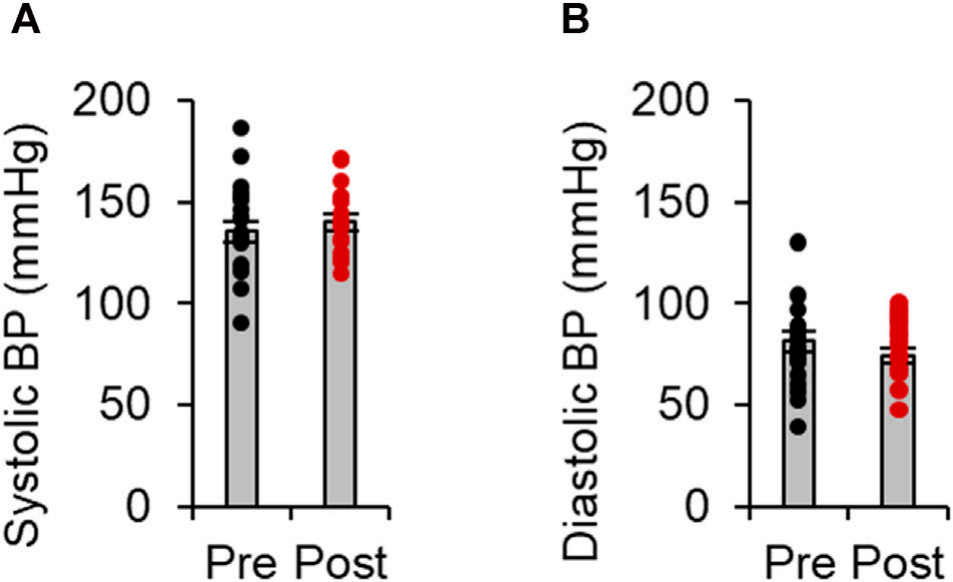
Effects of fasudil on blood pressure levels. Systolic **(A)** and diastolic **(B)** blood pressure (BP) before and after treatment with fasudil(n = 15). Data represent the mean ± s e.m.

Fasudil [30 mg, intravenous, twice daily (the maximum dose licensed for the treatment of subarachnoid hemorrhage in Japan)] was administered for an average of 12.9 ± 1.6 days. All patients tolerated the fasudil infusion well without any obvious side effects. As shown in Figures 2A,B, neither systolic nor diastolic blood pressure were significantly changed by fasudil treatment.

### Decreased Proteinuria in Patients Treated With Fasudil

**FIGURE 3:**
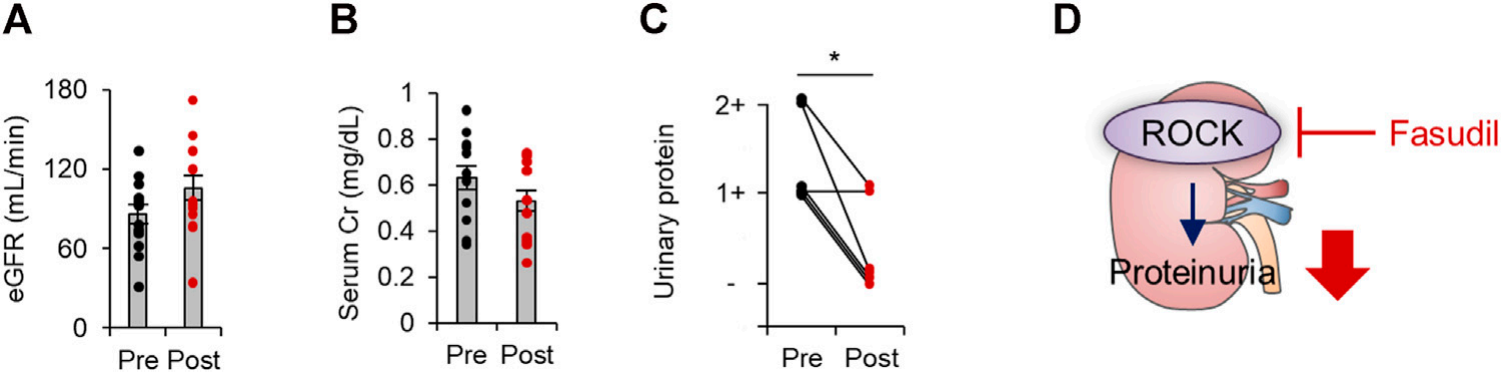
The renal outcomes in patients with diabetes treated with fasudil. **(A)** The estimated glomerular filtration rate (eGFR) before and after treatment with fasudil (*n* = 15). **(B)** Serum Cr levels before and after treatment with fasudil (*n* = 11). **(C)** The urinary protein levels evaluated by a dipstick test before and after treatment with fasudil (*n* = 6) *p < 0.05. Data represent the mean ± s.e.m. Cr, creatinine. **(D)** A schematic summary of key observations. In patients with diabetes, renal ROCK activity is elevated. Fasudil attenuates ROCK activity and proteinuria.

We finally evaluated the renal outcomes by assessing estimated glomerular filtration rate (eGFR), serum creatinine levels, and proteinuria before and after the treatment of fasudil in patients with diabetes. As shown in Figures 3A,B, a biochemical analysis revealed no significant changes in eGFR or serum creatinine levels. While physiological and biochemical examinations revealed no difference in blood pressure, eGFR, or serum creatinine levels from before to after fasudil treatment, we observed a significant reduction in proteinuria among patients with diabetes who received fasudil (Figure 3C). These data indicate that the pharmacological inhibition of ROCK could mitigate proteinuria in patients with diabetes.

## Discussion

Given the well-documented roles of ROCK as a pathogenic regulator of the onset and progression of diabetic nephropathy in rodents, we sought to discern whether patients with diabetic nephropathy exhibit heightened ROCK signaling. Using histological approaches, we detected elevated ROCK signaling in a broad area of renal tissue in the context of diabetes. This report is the first demonstration that renal ROCK is activated in patients with diabetes, which is in agreement with observations seen in the diabetic mouse kidney (Peng et al., 2008; Matoba et al., 2013). It is hypothesized that activated ROCK signaling contributes to the infiltration of macrophages into the glomeruli, the induction of fibrotic mediators (e.g., connective tissue growth factor, plasminogen activator inhibitor 1), and the production of reactive oxygen species in the diabetic kidney, which eventually leads to glomerular sclerosis (Kikuchi et al., 2007; Komers, 2011). We have extended these observations and shown that glomerular ROCK instigates the activation of transcriptional networks involving nuclear factor κB (NF-κB) and hypoxia-inducible factor 1α (HIF-1α) by accelerating the nuclear translocation and by inhibiting proteasome-dependent degradation, respectively (Matoba et al., 2013; Matoba et al., 2014). Collectively, these studies highlight the importance of the activated ROCK axis in facilitating glomerular damage in patients with diabetes.

It is noteworthy that the phosphorylated form of MYPT1 is strongly detected not only in glomeruli but also in the tubulointerstitial area. Previous studies revealed that ROCK is a key effector of tubulointerstitial fibrosis. For example, histological changes, collagen production, and extracellular matrix accumulation were attenuated by fasudil treatment in animal models of unilateral ureteral obstruction (Baba et al., 2015). However, the role of tubulointerstitial ROCK in diabetes remains to be elucidated. When the known pathological roles for ROCK in inflammation and fibrosis are taken into account, the observed tubular ROCK activation will subsequently impact on the downstream cellular cascades that induce tubulointerstitial damage in diabetes.

To date, two ROCK isoforms have been identified, ROCK1 (also known as Rho-kinase β/ROKβ) and ROCK2 (also referred to as Rho-kinase α/ROKα). These isoforms share 65% overall identity in amino acid sequence but are assumed to have distinct mechanisms of activation (Matoba et al., 2020): ROCK1 is activated by the cleavage of caspse-3, on the other hand, granzyme B is required for the activation of ROCK2. Recently, the unique functions of ROCK isoforms are beginning to be demonstrated. ROCK1 is reported to be involved in the mitochondrial biogenesis in podocytes (Wang et al., 2012) and in endothelial-to-mesenchymal transition (Peng et al., 2016), whereas mesangial ROCK2 is crucial for the cellular damage induced by transforming growth factor β (TGF-β) (Nagai et al., 2019). However, the distinctive and shared functions of ROCK isoforms are not completely understood. For future directions, it would be of interest to reveal isoform-specific roles in the development of diabetic nephropathy using cell-restricted gene manipulation approaches. Such delineation will pave the way for the development of ROCK isoform-specific inhibitors.

As stated above, we demonstrate that urinary protein excretion was decreased in patients treated with a ROCK inhibitor. Given that there were no significant changes in the blood pressure levels after fasudil treatment, this renoprotective effect seems to be independent of the systemic hemodynamics. Cell-based experiments revealed direct effect of ROCK inhibition on fibrotic reactions by attenuating renal inflammation and oxidative stress. This is concordant with previous observations obtained from experimental studies where better renal outcomes (e.g., histological improvement and mitigation in albuminuria) were demonstrated without affecting blood pressure levels (Matoba et al., 2013). Importantly, however, these findings are somewhat surprising in light of previous work showing a slight drop in blood pressure as a safety concern (Vicari et al., 2005). A number of considerations may account for the disparity in these findings. For instance, the incidence of hypotension in fasudil-treated subjects was only 0.4% in a multicenter post-marketing surveillance study (Suzuki et al., 2008). The phenotypic difference between humans and other animals should be also considered. Future studies aimed at elucidating the safety of fasudil in larger number of patients with diabetes, will hence prove beneficial.

The ROCK research field has been greatly expanded by the development of specific ROCK inhibitors (i.e., fasudil, hydroxy fasudil, and Y27632) (Ishizaki et al., 2000). These ATP-competitive small molecule inhibitors are widely used for basic research. Among these, fasudil is the only drug that is currently clinically available as an IV regimen. A large body of literature has shown the effectiveness of fasudil for the treatment of cerebral vasospasm. Based on the experimental and clinical evidence, fasudil is approved only for clinical use in the treatment of patients who have suffered subarachnoid hemorrhage. We therefore do note that all patients treated with fasudil in this study had subarachnoid hemorrhage, which might have affected the renal outcome by limiting their physical activity. In addition, blood glucose levels showed a decreasing trend after the treatment that will be explained by intensive diabetes care during hospitalization or the direct effect of fasudil. The major limitation of this study is the study population. The number of patients enrolled was too small to facilitate a proteinuria subgroup analysis (e.g., sex differences in the renoprotective effects of fasudil), although it was difficult to recruit subjects with these specific conditions. In addition, proteinuria determined by urinary dipstick may not fully represent an improvement in diabetic nephropathy. Although urinary albumin levels were not available in this retrospective study, the renoprotective effects of fasudil should be evaluated by analyzing the urinary albumin to creatinine ratio and in a large-scale prospective randomized study. To this end, future efforts are needed to develop an oral form of ROCK inhibitor that can be applied over longer periods. In addition, evaluation of renal histology will be of significant value to researchers and clinicians.

In conclusion, the current study identified elevated renal ROCK activity in patients with diabetic nephropathy. The patients with diabetes described in the present study tolerated fasudil well and proteinuria was attenuated without affecting their blood pressure levels. When considered alongside previous reports showing the roles of activated ROCK in renal inflammation and fibrosis, targeting this machinery may be a feasible approach for the treatment of diabetic nephropathy in humans.

## Data Availability

The raw data supporting the conclusion of this article will be made available by the authors, without undue reservation.

## References

[B1] Baba I. Egi Y. Utsumi H. Kakimoto T. Suzuki K. (2015). Inhibitory effects of fasudil on renal interstitial fibrosis induced by unilateral ureteral obstruction. Mol. Med. Rep. 12 (6), 8010–8020. 10.3892/mmr.2015.4467 26498136 PMC4758322

[B2] Brosius F. C. Tuttle K. R. Kretzler M. (2016). JAK inhibition in the treatment of diabetic kidney disease. Diabetologia 59 (8), 1624–1627. 10.1007/s00125-016-4021-5 27333885 PMC4942738

[B3] Cahn A. Cernea S. Raz I. (2019). The SONAR study-is there a future for endothelin receptor antagonists in diabetic kidney disease?. Ann. Transl Med. 7 (Suppl. 8), S330. 10.21037/atm.2019.09.117 32016048 PMC6976431

[B4] de Zeeuw D. Heerspink H. J. L. (2016). Unmet need in diabetic nephropathy: failed drugs or trials?. Lancet Diabetes Endocrinol. 4 (8), 638–640. 10.1016/S2213-8587(16)30045-6 27160545

[B5] Fujisawa K. Fujita A. Ishizaki T. Saito Y. Narumiya S. (1996). Identification of the Rho-binding domain of p160ROCK, a Rho-associated coiled-coil containing protein kinase. J. Biol. Chem. 271 (38), 23022–23028. 10.1074/jbc.271.38.23022 8798490

[B6] Gojo A. Utsunomiya K. Taniguchi K. Yokota T. Ishizawa S. Kanazawa Y. (2007). The Rho-kinase inhibitor, fasudil, attenuates diabetic nephropathy in streptozotocin-induced diabetic rats. Eur. J. Pharmacol. 568 (1-3), 242–247. 10.1016/j.ejphar.2007.04.011 17511984

[B7] Heerspink H. J. L. Kohan D. E. de Zeeuw D. (2021). New insights from SONAR indicate adding sodium glucose co-transporter 2 inhibitors to an endothelin receptor antagonist mitigates fluid retention and enhances albuminuria reduction. Kidney Int. 99 (2), 346–349. 10.1016/j.kint.2020.09.026 33144213

[B8] Ishizaki T. Uehata M. Tamechika I. Keel J. Nonomura K. Maekawa M. (2000). Pharmacological properties of Y-27632, a specific inhibitor of rho-associated kinases. Mol. Pharmacol. 57 (5), 976–983. 10779382

[B9] Kanasaki K. Nagai T. Nitta K. Kitada M. Koya D. (2014). N-acetyl-seryl-aspartyl-lysyl-proline: a valuable endogenous anti-fibrotic peptide for combating kidney fibrosis in diabetes. Front. Pharmacol. 5, 70. 10.3389/fphar.2014.00070 24782774 PMC3995071

[B10] Kikuchi Y. Yamada M. Imakiire T. Kushiyama T. Higashi K. Hyodo N. (2007). A Rho-kinase inhibitor, fasudil, prevents development of diabetes and nephropathy in insulin-resistant diabetic rats. J. Endocrinol. 192 (3), 595–603. 10.1677/JOE-06-0045 17332527

[B11] Komers R. Oyama T. T. Beard D. R. Tikellis C. Xu B. Lotspeich D. F. (2011). Rho kinase inhibition protects kidneys from diabetic nephropathy without reducing blood pressure. Kidney Int. 79 (1), 432–442. 10.1097/MNH.0b013e32834131f810.1038/ki.2010.428 20962741

[B12] Li J. Liu H. Srivastava S. P. Hu Q. Gao R. Li S. (2020). Endothelial FGFR1 (Fibroblast Growth Factor Receptor 1) Deficiency Contributes Differential Fibrogenic Effects in Kidney and Heart of Diabetic Mice. Hypertension 76 (6), 1935–1944. 10.1161/HYPERTENSIONAHA.120.15587 33131311

[B13] Lytvyn Y. Godoy L. C. Scholtes R. A. van Raalte D. H. Cherney D. Z. (2019). Mineralocorticoid Antagonism and Diabetic Kidney Disease. Curr. Diab Rep. 19 (1), 4. 10.1007/s11892-019-1123-8 30673886

[B14] Masumoto A. Mohri M. Shimokawa H. Urakami L. Usui M. Takeshita A. (2002). Suppression of coronary artery spasm by the Rho-kinase inhibitor fasudil in patients with vasospastic angina. Circulation 105 (13), 1545–1547. 10.1161/hc1002.105938 11927519

[B15] Matoba K. Kawanami D. Okada R. Tsukamoto M. Kinoshita J. Ito T. (2013). Rho-kinase inhibition prevents the progression of diabetic nephropathy by downregulating hypoxia-inducible factor 1α. Kidney Int. 84 (3), 545–554. 10.1038/ki.2013.130 23615507

[B16] Matoba K. Kawanami D. Tsukamoto M. Kinoshita J. Ito T. Ishizawa S. (2014). Rho-kinase regulation of TNF-α-induced nuclear translocation of NF-κB RelA/p65 and M-CSF expression via p38 MAPK in mesangial cells. Am. J. Physiol. Ren. Physiol 307 (5), F571–F580. 10.1152/ajprenal.00113.2014 25007875

[B17] Matoba K. Takeda Y. Nagai Y. Sekiguchi K. Yokota T. Utsunomiya K. (2020). The Physiology, Pathology, and Therapeutic Interventions for ROCK Isoforms in Diabetic Kidney Disease. Front. Pharmacol. 11, 585633. 10.3389/fphar.2020.585633 33101039 PMC7545791

[B18] Nagai Y. Matoba K. Kawanami D. Takeda Y. Akamine T. Ishizawa S. (2019). ROCK2 regulates TGF-β-induced expression of CTGF and profibrotic genes via NF-κB and cytoskeleton dynamics in mesangial cells. Am. J. Physiol. Ren. Physiol 317 (4), F839–F851. 10.1152/ajprenal.00596.2018 31364374

[B19] Peng F. Wu D. Gao B. Ingram A. J. Zhang B. Chorneyko K. (2008). RhoA/Rho-kinase contribute to the pathogenesis of diabetic renal disease. Diabetes 57 (6), 1683–1692. 10.2337/db07-1149 18356410

[B20] Peng H. Li Y. Wang C. Zhang J. Chen Y. Chen W. (2016). ROCK1 Induces Endothelial-to-Mesenchymal Transition in Glomeruli to Aggravate Albuminuria in Diabetic Nephropathy. Sci. Rep. 6, 20304. 10.1038/srep20304 26842599 PMC4740844

[B21] Shimokawa H. (2002). Rho-kinase as a novel therapeutic target in treatment of cardiovascular diseases. J. Cardiovasc. Pharmacol. 39 (3), 319–327. 10.1097/00005344-200203000-00001 11862109

[B22] Srivastava S. P. Goodwin J. E. Kanasaki K. Koya D. (2020). Inhibition of Angiotensin-Converting Enzyme Ameliorates Renal Fibrosis by Mitigating DPP-4 Level and Restoring Antifibrotic MicroRNAs. Genes (Basel) 11 (2), 211. 10.3390/genes11020211 PMC7074526 32085655

[B23] Srivastava S. P. Li J. Kitada M. Fujita H. Yamada Y. Goodwin J. E. (2018). SIRT3 deficiency leads to induction of abnormal glycolysis in diabetic kidney with fibrosis. Cell Death Dis 9 (10), 997. 10.1038/s41419-018-1057-0 30250024 PMC6155322

[B24] Srivastava S. P. Li J. Takagaki Y. Kitada M. Goodwin J. E. Kanasaki K. (2021a). Endothelial SIRT3 regulates myofibroblast metabolic shifts in diabetic kidneys. iScience 24 (5), 102390. 10.1016/j.isci.2021.102390 33981977 PMC8086030

[B25] Srivastava S. P. Zhou H. Setia O. Liu B. Kanasaki K. Koya D. (2021b). Loss of endothelial glucocorticoid receptor accelerates diabetic nephropathy. Nat. Commun. 12 (1), 2368. 10.1038/s41467-021-22617-y 33888696 PMC8062600

[B26] Suzuki Y. Shibuya M. Satoh S. Sugiyama H. Seto M. Takakura K. (2008). Safety and efficacy of fasudil monotherapy and fasudil-ozagrel combination therapy in patients with subarachnoid hemorrhage: sub-analysis of the post-marketing surveillance study. Neurol. Med. Chir (Tokyo) 48 (6), 241–248. discussion 247-248. 10.2176/nmc.48.241 18574328

[B27] Takeda Y. Matoba K. Kawanami D. Nagai Y. Akamine T. Ishizawa S. (2019). ROCK2 Regulates Monocyte Migration and Cell to Cell Adhesion in Vascular Endothelial Cells. Int. J. Mol. Sci. 20 (6), 1331. 10.3390/ijms20061331 PMC6471293 30884801

[B28] Uehata M. Ishizaki T. Satoh H. Ono T. Kawahara T. Morishita T. (1997). Calcium sensitization of smooth muscle mediated by a Rho-associated protein kinase in hypertension. Nature 389 (6654), 990–994. 10.1038/40187 9353125

[B29] Vicari R. M. Chaitman B. Keefe D. Smith W. B. Chrysant S. G. Tonkon M. J. (2005). Efficacy and safety of fasudil in patients with stable angina: a double-blind, placebo-controlled, phase 2 trial. J. Am. Coll. Cardiol. 46 (10), 1803–1811. 10.1016/j.jacc.2005.07.047 16286163

[B30] Waijer S. W. Xie D. Inzucchi S. E. Zinman B. Koitka-Weber A. Mattheus M. (2020). Short-Term Changes in Albuminuria and Risk of Cardiovascular and Renal Outcomes in Type 2 Diabetes Mellitus: A Post Hoc Analysis of the EMPA-REG OUTCOME Trial. J. Am. Heart Assoc. 9 (18), e016976. 10.1161/JAHA.120.016976 32893717 PMC7727012

[B31] Wang W. Wang Y. Long J. Wang J. Haudek S. B. Overbeek P. (2012). Mitochondrial fission triggered by hyperglycemia is mediated by ROCK1 activation in podocytes and endothelial cells. Cell Metab 15 (2), 186–200. 10.1016/j.cmet.2012.01.009 22326220 PMC3278719

[B32] Wanner C. Inzucchi S. E. Lachin J. M. Fitchett D. von Eynatten M. Mattheus M. (2016). Empagliflozin and Progression of Kidney Disease in Type 2 Diabetes. N. Engl. J. Med. 375 (4), 323–334. 10.1056/NEJMoa1515920 27299675

[B33] Wickman G. Lan C. Vollrath B. (2003). Functional roles of the rho/rho kinase pathway and protein kinase C in the regulation of cerebrovascular constriction mediated by hemoglobin: relevance to subarachnoid hemorrhage and vasospasm. Circ. Res. 92 (7), 809–816. 10.1161/01.RES.0000066663.12256.B2 12637369

[B34] Zhao J. Zhou D. Guo J. Ren Z. Zhou L. Wang S. (2011). Efficacy and safety of fasudil in patients with subarachnoid hemorrhage: final results of a randomized trial of fasudil versus nimodipine. Neurol. Med. Chir (Tokyo) 51 (10), 679–683. 10.2176/nmc.51.679 22027241

