# Supplementary material for: Renal ROCK Activation and Its Pharmacological Inhibition in Patients With Diabetes
Source: Front Pharmacol. 2021 Sep 7;12:738121. doi: 10.3389/fphar.2021.738121 (PMC8454778; doi:10.3389/fphar.2021.738121)
Supplement: Supplementary file 1 [file Table1.pdf]

Tissue type	Age (y)	Sex	BMI (kg/m^2)	PMI (h)	Cause of death	Comorbidities
Normal #1	62	M	24.5	3	Congestive heart failure	Alcoholism
Normal #2	68	M	24.5	5	Necrotic colitis	Coronary artery disease
Normal #3	52	M	27.7	4.5	Gastric perforation	Thyroid nodule
Normal #4	50	M	35.4	4	Acute coronary syndrome	Atherosclerosis
Diabetic nephropathy #1	62	M	26.3	3	Rupture of aortic aneurism	Atherosclerosis
Diabetic nephropathy #2	48	M	50.5	4	Gastric perforation	Metabolic syndrome
Diabetic nephropathy #3	56	F	27.7	5.5	Stroke	Atherosclerosis
Diabetic nephropathy #4	90	M	24.8	3	Congestive heart failure	Hypertension

SUPPLEMENTAL TABLE 1 | Clinical information of human kidney samples used for the histological examination. The patients' pathological diagnosis, age at the time of death, sex, BMI, PMI, cause of death, and comorbidities are shown. BMI, body mass index; PMI, postmortem interval.
