# Supplementary figures and images for: Renal ROCK Activation and Its Pharmacological Inhibition in Patients With Diabetes
Source: Front Pharmacol. 2021 Sep 7;12:738121. doi: 10.3389/fphar.2021.738121 (PMC8454778; doi:10.3389/fphar.2021.738121)

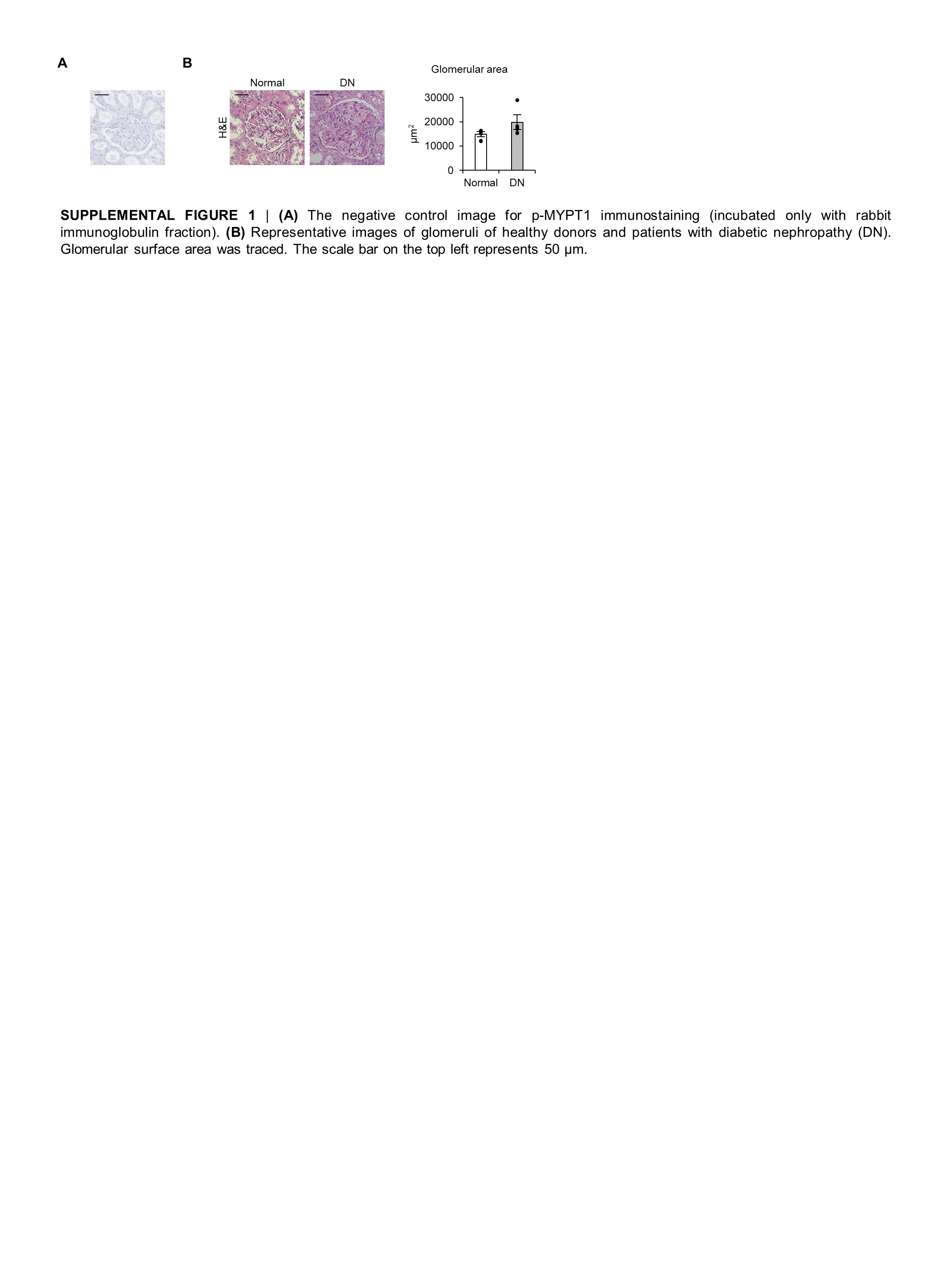

Supplement: Supplementary file 2 [file Image1.tif]
